# Performance of Proximity Loggers in Recording Intra- and Inter-Species Interactions: A Laboratory and Field-Based Validation Study

**DOI:** 10.1371/journal.pone.0039068

**Published:** 2012-06-26

**Authors:** Julian A. Drewe, Nicola Weber, Stephen P. Carter, Stuart Bearhop, Xavier A. Harrison, Sasha R. X. Dall, Robbie A. McDonald, Richard J. Delahay

**Affiliations:** 1 Royal Veterinary College, Hawkshead Lane, North Mymms, Herts, United Kingdom; 2 Centre for Ecology and Conservation, College of Life and Environmental Sciences, University of Exeter, Cornwall Campus, Penryn, United Kingdom; 3 The Food and Environment Research Agency, Sand Hutton, York, United Kingdom; 4 Environment and Sustainability Institute, College of Life and Environmental Sciences, University of Exeter, Cornwall Campus, Penryn, United Kingdom; Cajal Institute, Consejo Superior de Investigaciones Científicas, Spain

## Abstract

Knowledge of the way in which animals interact through social networks can help to address questions surrounding the ecological and evolutionary consequences of social organisation, and to understand and manage the spread of infectious diseases. Automated proximity loggers are increasingly being used to record interactions between animals, but the accuracy and reliability of the collected data remain largely un-assessed. Here we use laboratory and observational field data to assess the performance of these devices fitted to a herd of 32 beef cattle (*Bos taurus)* and nine groups of badgers (*Meles meles*, *n*  = 77) living in the surrounding woods. The distances at which loggers detected each other were found to decrease over time, potentially related to diminishing battery power that may be a function of temperature. Loggers were highly accurate in recording the identification of contacted conspecifics, but less reliable at determining contact duration. There was a tendency for extended interactions to be recorded as a series of shorter contacts. We show how data can be manipulated to correct this discrepancy and accurately reflect observed interaction patterns by combining records between any two loggers that occur within a 1 to 2 minute amalgamation window, and then removing any remaining 1 second records. We make universally applicable recommendations for the effective use of proximity loggers, to improve the validity of data arising from future studies.

## Introduction

Interactions between animals influence a broad array of social processes [1], and researchers may be interested in quantifying patterns of interactions to address important behavioural, ecological and evolutionary questions. Examples include studies of the spread of information and infectious diseases [2]. However, empirical data on interactions between individuals are sparse, particularly in free-ranging wild animals [3]. Methods employed in previous studies have relied on either direct observation of contact between individuals [4], [5] or the ability to infer contact using proxy measures of shared space from data collected by methods such as radio-telemetry and Global Positioning System (GPS) locations [6], [7]. Such methods of directly tracking individual animals to record their interactions are expensive, time consuming, and limited to animals that are readily and easily observable from a distance, or to species that habituate quickly to the presence of observers. Also, these data often lack fine-scale spatial resolution. Using telemetry to automatically collect animal interaction data will enable more refined studies of larger groups of animals.

One increasingly popular method is the use of proximity detectors (e.g. proximity data logger systems, Sirtrack Tracking Solutions, Havelock North, New Zealand). These remote-sensing devices are attached to animals via collars, harnesses or ear tags, or in some cases they may be glued directly on to the animal e.g. seals and hedgehogs. They transmit a unique signal and automatically record frequency and duration of contacts when tagged animals come within a pre-set distance of one another. Proximity loggers have been used in a small number of focussed animal studies; however, they have the potential to address a broader range of behavioural, ecological and evolutionary questions. Proximity logging devices have been employed in several studies of wild and domestic animals including contact networks in captive brushtail possums *Trichosurus vulpecula* [8]; proximity detection in wild raccoons *Procyon lotor* [9]; cow-cow, cow-calf and ewe-lamb interactions in domestic livestock [10], [11], [12]; contact rates between Eurasian badgers *Meles meles* [13] and between badgers and cattle [14]; population network structure of wild Tasmanian devils *Sarcophilus harrisii* [15]; and in revealing spatial and temporal heterogeneity in the behaviour of European rabbits *Oryctolagus cuniculus* [16]. Proximity loggers provide data that can be used to develop quantitative contact networks, which may offer insights into behavioural and social processes, and can potentially lead to improvements in disease management [17], [18].

Despite enthusiastic adoption of this novel technology, the accuracy and reliability of data collected by proximity loggers are often unmeasured (but see [9], [13], [19]). Proximity-logging devices have several user-defined parameters, including the receiver power which is a proxy for distance at which contacts are detected, making them amenable to investigations with different study species and different objectives. An earlier study investigated the performance of a prototype version in the laboratory and on raccoons in the field and reported a 43% failure rate [9]. Whilst recent changes to the radio chip should enable the proximity loggers to perform better, data collected by such devices have often been used without explicit validation. Ultimately, complete precision is not possible as radio waves can be reflected, refracted and/or absorbed by naturally occurring compounds, including natural features such as vegetation, water bodies and terrain [20]. There is a need to explore data processing methods that quantify and minimise errors associated with the use of proximity loggers. Various data processing methods (described below) have been used [9], [15], [16], but their widespread applicability has yet to be investigated. In particular, in previous studies proximity loggers interacting at the edge of their detection range have been shown to frequently record very short contacts (typically of 1 second duration), thought to be due to weak signal strength [9]. Removing these records from the dataset has been reported to increase the reliability of dyadic contact records [9], but may have profound effects on the structure of contact networks calculated from frequency data [15]. If data removal is conducted after any broken records have been combined (see methods), then this could further improve the accuracy of the recorded data in terms of how they reflect ‘true’ patterns of interaction.

The performance of proximity loggers in recording interspecies contacts has yet to be validated. It is important that the data collected by proximity loggers are closely examined and calibrated against simultaneous observations before conclusions are drawn. Also, as the technology improves, it is likely that proximity loggers will become smaller and less expensive, and so will become more widely adopted in studies of the social behaviours of wild animals. It is important that unified methods for data collection, filtering and analyses are tested, refined and adopted. The aim of this research was to perform a validation study using data collected in both the laboratory and the field to validate the information gathered by proximity loggers attached via collars to cattle and badgers, and on static base stations in the field. Investigating contact patterns in this system is of particular contemporary interest because of the role of the badger in the perpetuation of bovine tuberculosis (bTB) in cattle herds in the UK and Ireland [21]. We use our findings to make universally applicable recommendations for the effective use of proximity loggers in future studies of animal interactions.

## Materials and Methods

### Study Location and Species

This study was undertaken over 18 months from April 2009 to September 2010 at Woodchester Park, Gloucestershire, UK (51°71′N, 2°30′W). This is a 7 km^2^ region of Cotswold limestone escarpment consisting of a wooded valley with areas of pasture grazed by a herd of approximately 35 Welsh Black cattle. The site also contains an intensively studied population of 200–300 wild badgers belonging to 24 different social groups with a mean size of 10. This badger population has been the subject of long-term ecological and epidemiological research and their territorial organisation and the methods employed for their capture are well described [22].

### Equipment Deployed

Three configurations of the same proximity logger were used in this study: badger collars (*n*  = 77), cattle collars (*n*  = 32), and static base stations (*n*  = 19). All were manufactured by Sirtrack Tracking Solutions (Havelock North, New Zealand), and differed in packaging but operated in the same manner using the same hardware (although the badger collars also included a Very High Frequency (VHF) transmitter, see below). Proximity data-logging collars consist of an Ultra High Frequency (UHF) transceiver that broadcasts a unique ID code, whilst simultaneously 'listening' for those of others. When two or more units come within a pre-determined, user-defined distance (see individual sections below for details), a contact is initiated until one or both of the receiving loggers fails to detect the signal within a user-defined separation time. Collars were set to have a separation time of 10 seconds, meaning that a single continuous encounter would be recorded until the receiving logger(s) failed to detect the transmitting logger’s signal for a period longer than 10 seconds. At this time, each receiving unit logs the date, starting time and the duration of the interaction with the other unit(s). Interaction data stored in the loggers were periodically downloaded onto a laptop computer using the supplied interface and software.

#### Badger proximity loggers

**Figure 1:**
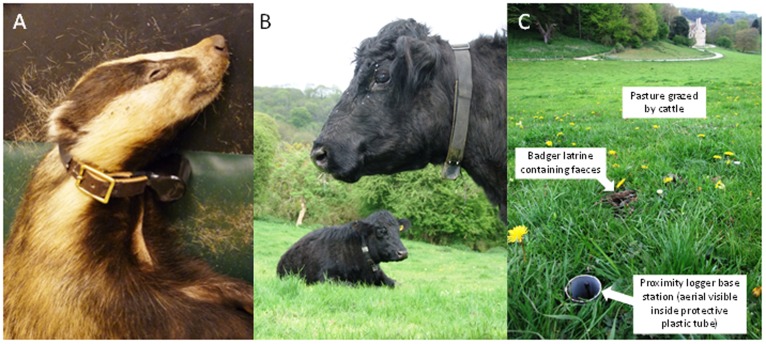
The three types of proximity logger used in this study. (A) Proximity logger on a collar fitted to an anaesthetised badger. (B) Cattle wearing proximity logger collars. (C) Proximity logger base station in situ near a badger latrine in a field grazed by the collared cattle.

77 badgers from nine social groups were fitted with proximity loggers on adjustable leather collars whilst under anaesthesia (Fig. 1A). These collars remained on the badgers for up to 17 months from May 2009 to September 2010 with 70% of collars continuously recording data for more than 9 months. Proximity loggers on animals that stay close to the ground, such as badgers, have a shorter expected transmission distance than those on animals of a greater height, such as cattle. We trialled two methods for setting the detection range of the badger proximity loggers: (i) all collars set to the same UHF setting which was selected through a trial to minimise the variation in detection distances across all collars [*same setting*] and (ii) each collar individually set to a collar-specific UHF setting that resulted in the same detection distance as the rest of the collars [*individual setting*]. For the *same setting* study, the detection range of 16 badger proximity loggers was set at UHF 37, which in the trial of randomly paired collars conducted over a range of distances was found to equate to a contact initiation distance of 0.77±0.27 m (mean ± s.d.) and a contact termination distance of 0.93±0.36 m (Table 1). For the *individual setting* study, the remaining 61 badger proximity collars were individually set using across a range of UHF power settings (range: UHF 34 to UHF 48) that resulted in a contact initiation distance of 0.64±0.04 m and a termination distance of 0.87±0.11 m (Table 1). These short-range detection distances were chosen to record direct contacts between collared badgers such as bite-wounding and grooming, as well as to be within the likely aerosol transmission distance for *Mycobacterium bovis* (the causative agent of bTB) [23], [24]. Each badger proximity logger collar also emitted a VHF radio signal that allowed for the animals (or collars that had been shed by animals) to be located in the field using standard radio tracking methods.

**Table 1:** Changes in the detection distances of proximity loggers over time in the field.

Interaction Type	UHF Setting	Time (months after deployment)	n	Initiation Distance (m)			Termination Distance (m)		
				Mean (sd)	Min	Max	Mean (sd)	Min	Max
Cattle – Cattle	45	0	32	1.70 (0.12)	1.47	1.94	1.92 (0.14)	1.62	2.24
		15	29	1.29 (0.30)	0.85	1.80	1.51 (0.41)	0.95	2.40
		*% change in detection distance*		−*24 (145)*	−*42*	−*7*	−*21 (180)*	−*41*	*7*
Badger – Badger	37 (*same setting*)	0	15	0.77 (0.27)	0.40	1.40	0.93 (0.36)	0.65	1.80
		8	10	0.38 (0.16)	0.10	0.60	0.49 (0.21)	0.10	0.70
		*% change in detection distance*		−*51 (39)*	−*75*	−*57*	−*48 (41)*	−*85*	−*61*
	34–48 (*individual setting*)	0	61	0.64 (0.04)	0.57	0.71	0.87 (0.11)	0.70	1.11
		12	20	0.32 (0.03)	0.25	0.37	0.58 (0.06)	0.49	0.70
		*% change in detection distance*		−*50 (25)*	−*56*	−*48*	−*33 (45)*	−*30*	−*37*
		17	20	0.31 (0.05)	0.24	0.39	0.60 (0.05)	0.46	0.71
		*% change in detection distance*		−*52 (25)*	−*58*	−*45*	−*31 (55)*	−*34*	−*36*
Cattle – Badger		20	10	1.33 (0.57)	0.20	2.28	1.57 (0.90)	0.25	3.32
Badger – Cattle		20	10	1.22 (0.63)	0.20	2.28	1.49 (0.76)	0.25	2.75
Base Station – Base Station		0	14	0.55 (0.13)	30	75	NR	NR	NR
		4	12	0.47 (0.15)	20	60	NR	NR	NR
		*% change in detection distance*		−*15 (14)*	−*33*	−*20*	NR	NR	NR

NR  =  not recorded.

Loggers were deployed on collars fitted to cattle and badgers, and in static base stations, for up to 17 consecutive months from May 2009 to September 2010. Initiation distance refers to the distance between loggers when a contact starts. Termination distance refers to the distance between loggers when a contact ends. Changes in logger detection distances over the course of the study are given in italics; negative values indicate a reduction in detection distance over time.

#### Cattle proximity loggers

32 cattle were fitted with proximity loggers on adjustable collars made from synthetic belting (Fig. 1B). Collars were fitted in September 2009 and remained on for 12 months, although not all collars recorded data over the whole time period due to the logger memories reaching maximum capacity (16,384 records) before they could be downloaded. Cattle collars were set to a detection range of UHF 45 (using a similar method to the *same setting* badger collars), which in the laboratory trial between pairs of cattle collars was found to equate to a contact initiation distance of 1.70±0.12 m and a contact termination distance of 1.92±0.14 m (*n*  = 32 collars: Table 1). Thus a cattle collar on a focal cow should have detected other cattle collars, and badger collars, if any came within 1.92 m of the focal cow. This distance is likely to be biologically meaningful in the epidemiology of bTB because it approximates the 1.5–2.0 m aerosol transmission distance postulated to occur between cattle and possums [23], [24]. Aerosol transmission is considered to be one of the more important transmission routes for *M. bovis* between wildlife and cattle [25].

#### Static base stations

Nineteen static base stations were submerged in plastic tubes next to badger latrines located on the pasture grazed by the cattle (Fig. 1C). Badger latrines may represent potentially important sources of environmental exposure to *M. bovis* in badger faeces and urine [26]. In addition, badgers use these communal latrines to demarcate territorial boundaries, and so they are likely to represent nodes of interaction amongst individuals from neighbouring social groups. Base stations were set to UHF 20 (using the *same setting* method), which in a trial with base stations placed in tubes in the ground was found to equate to a contact initiation distance of 0.55±0.13 m (Table 1). This relatively short detection distance is likely to be related to the position of the loggers buried in the ground (Fig. 1C) which may absorb or deflect radio waves [20]. Static base stations were deployed for up to 4.5 months between April and September 2010.

### Validation in the Laboratory

#### Proximity logger detection distances and variation over time

To ascertain the distance over which proximity loggers recorded interactions at different UHF settings, loggers were subjected to a laboratory-based trial. Badger collars were attached to 2-litre plastic bottles filled with saline to mimic UHF wave absorption that would occur when worn by the animal (K. Lay, *pers comms*). Cattle collars were held by a person rather than being attached to saline-filled bottles. Collars were positioned at 0 cm, 10 cm and 100 cm above ground level, representing static, badger and cattle collar positioning respectively. The badger collars, cattle collars and static base stations were randomly allocated into same-type pairs and placed 3 m apart on the ground next to an extended tape measure. Within each pair, one logger was moved towards the other in 1 cm increments every 20 seconds until the illuminated LED indicated the two loggers had established a contact. The LED was turned off when deployed on the free-ranging animals so as not to disrupt normal behaviours. This separation distance was recorded, being the contact initiation distance for the first logger. The distance between the pair of loggers was further reduced until the second logger detected the first. The loggers were then gradually moved apart until a long LED pulse indicated one logger had lost contact with the other (this was recorded as the contact termination distance for first logger). The distance was further increased until the second logger lost contact with the first (the contact termination distance for the second logger).

In a test to mimic inter-species contacts, 10 cattle collars and 10 badger collars were randomly allocated into pairs to investigate initiation and termination distances. In each trial the cattle collar was held 1 m above the ground and the badger collar 10 cm above the ground, and collars were moved towards each other using the same protocol detailed above. The initiation and termination distances were calculated as the hypotenuse of a right-angled triangle formed from the horizontal and vertical distances between the interacting cattle and badger collars.

To establish whether detection ranges remained constant over time, we used the same laboratory-based method to compare contact initiation and termination distances at various stages during the study (8, 12 and 17 months post-deployment) with those recorded prior to deployment, for all types of logger. In addition, at the end of the 17 months, two cattle collars that had not been deployed (but were the same age as those that had been on cattle in the field) were tested to determine their contact initiation and termination distances so that findings could be related to battery charge. Changes in initiation and termination distances were tested against the frequency and duration of contacts recorded by the collars.

#### Broken contacts

A previously identified limitation of the proximity logger technology is the tendency for a continuous contact to be recorded as a series of multiple shorter contacts [9]. If these data are analysed without correction for this phenomenon then results and conclusions concerning the frequency and duration of interactions are likely to be misleading. A laboratory trial was undertaken whereby 25 pairs of badger proximity loggers were attached to 2-litre bottles of saline and placed facing each other at 0.30 m apart for 2 hours. As they were set to a contact initiation distance of 0.64 m (see above), they were well-within detection range of one another, and theoretically should have recorded the encounter as one continual contact of 2 hours (7,200 seconds) duration. If a break in the contact recording occurred, the time difference between the end of the broken contact and the initiation of the next contact was calculated and then averaged a) for each collar individually to assess intra-unit variation, and b) for all collars together in order to give an overall value that could be used as a threshold for combining the broken records into a continuous contact.

In previous studies, proximity loggers interacting at the edge of their detection range have been shown to often record very short contacts (typically of 1 second duration), thought to be due to weak signal strength [9], [15]. Removing these records from the dataset has been reported to increase the reliability of dyadic contact records [9]. If they were removed after any broken records have been combined (based on the threshold calculated above) then this could further improve the accuracy of the recorded data in terms of how they reflect the ‘true’ interactions We investigated the effect of omitting 1 second records from the proximity logger dataset post-amalgamation on the dataset’s similarity to the observational data.

#### Reciprocal contacts

To determine the accuracy of proximity loggers at correctly recording identification codes of other loggers, the databases of all recorded interactions for all three types of device were examined. To determine if the reliability of data varied between badger proximity loggers set using *same setting* or *individual setting* UHF settings, and therefore determine the necessity of setting each collar individually, we compared the frequency and duration of reciprocal records between five pairs of loggers in each of the three possible UHF setting combinations (*same setting-same setting, individual setting-same setting, individual setting-individual setting*) collected in the field during one calendar month (June 2010). For each pairing, a linear regression was performed on the log-transformed values for collar 1 against collar 2, for both frequency (number) and duration of contacts. The residual values were then compared using a one-way ANOVA to determine whether they varied significantly between the three different pairings in frequency and duration of shared contacts. This analysis was conducted three times using the statistical software R [27]: first, using the data exactly as recorded on the proximity loggers; second, after manipulating the dataset to amalgamate dyadic records occurring within 1 min of each other (this being approximately the median gap duration for broken contacts: see Results); and third, after amalgamation followed by removal of any remaining contact records lasting 1 second (see above).

### Validation in the Field

#### Cattle observation study

To validate the data collected by the cattle proximity loggers, focal observations of interactions between collared cattle were conducted in the field by an observer over two days in June 2010. Twelve randomly-selected cattle were each observed for 30 minutes from a distance of approximately 20 m. Cattle were considered to be interacting with each other if they were within one head’s width of the other animal: this ensured that they were within the mean contact initiation distance to which the cattle collars were set (1.7 m). All interactions were recorded during each 30 minute focal period, noting the identification of the partner (read from ear tag number using binoculars), the start and end time of the contact, and the type of interaction (e.g. grooming, head butting, walking by etc.). Observational data were compared to those recorded by the collars to determine the accuracy of the loggers in recording number of contacts, duration of contacts and contacted logger identification. Paired t-tests were used to test for differences between observed and recorded data.

## Results

### Logger and Data Retrieval

Of the 77 badger collars fitted: 28 (36%) were retrieved by re-trapping the badgers; 25 (33%) were retrieved by locating the dropped collar in the field using radio-telemetry (in the majority of cases this was due to a snapped collar); seven (9%) were lost (no VHF signal detectable); six (8%) had fallen off underground and could not be retrieved; and 11 (14%) were still fitted on badgers at the time of writing. Of the 32 cattle collars that were deployed at the start of the study, 29 loggers (91%) were recovered undamaged (although the collar strapping of one was broken) and three loggers were lost (collars fell off but were not found). Of the 19 base stations used in the study, 11 (58%) were recovered and eight went missing (presumed to have been dug up and removed by badgers).

### Validation in the Laboratory

#### Proximity logger detection distances and variation over time

All three types of logger showed a reduction in their detection range over time (Table 1). The largest reduction was seen in the badger collars where logger initiation distances reduced by 50% within 8 months of deployment, but then stayed constant at this decreased value for the next 9 months (Table 1). In addition, badger loggers showed a pronounced reduction in mean termination distance and a shortening of the range of detection distances over this time (indicated by the decrease in standard deviations for initiation and termination distances: Table 1). Cattle collars showed a moderate reduction in mean initiation and termination distances and a widening of the range of detection distances over the study period (indicated by the increase in standard deviations for initiation and termination distances: Table 1). However, the two cattle collars that were tested after this time that had not been deployed in the field and were stored with their batteries turned off did not show any decrease. The base stations, although tested over a shorter time period than the collars, still showed an overall reduction in mean contact detection distance from 0.55±0.13 m to 0.47±0.15 m during the study period. For the badger and cattle collars, the decreases in detection distances were not found to be influenced by the number (F_1, 60_ = 1.17, *P*  = 0.30) or by the duration (F_1, 60_ = 2.37, *P*  = 0.13) of contacts that they had recorded during deployment in the field.

Cattle and badger collars were tested against each other at different heights to mimic interspecific contacts. The detection distances were found to have a wider range than for the same collars detecting intraspecific contacts (Table 1). However, despite the two types of collar having different UHF settings, there was very little difference in the detection ranges for each type of collar when detecting the other (Table 1).

#### Broken contacts

In none of the laboratory trials of 25 pairs of badger collars was the contact recorded as a continuous 2-hour interaction, but rather always as a series of multiple broken contacts. Intra-collar variation was found to be minimal, and across all 50 collars, the median gap duration between the end of one recorded contact and the initiation of the next was 54 s (range: 28 to 628 s; mode: 47 s), and the 95^th^ percentile gap duration was 129 s. See below for field validation of broken contacts.

#### Reciprocal contacts

**Figure 2:**
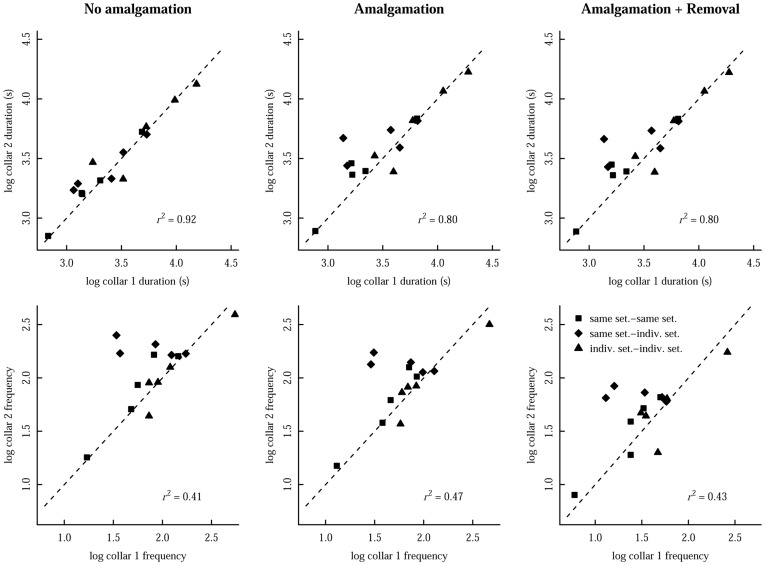
Correlations between contacts recorded by interacting pairs of badger proximity collars. Values are given for a) the duration and b) the frequency (number) of contacts for the 3 possible collar pairings based on their UHF settings (*indiv. set.-indiv. set.* (▴), *indiv. set.-same set.* (♦), *same set.-same set.* (▪)) and for the three data manipulation treatments to reflect the ‘real-life’ contacts (no amalgamation of broken contacts, amalgamation, amalgamation and removal of remaining 1 second contacts). The dashed line is the line of equivalence (y  =  x), along which all points would lie if collar 1 recorded exactly the same data as collar 2.

There was a high level of agreement in the durations of the contacts recorded by one collar and the reciprocal interacting collar under all three treatment scenarios: no amalgamation of contacts (F_1,14_ = 155.0, *P*<0.001, *r* ^2^ = 0.92); amalgamation of those less than 1 minute apart (F_1,14_ = 49.4, *P*<0.001, *r* ^2^ = 0.80); and amalgamation and removal of any remaining 1 second contacts (F_1,14_ = 50.5, *P*<0.001, *r* ^2^ = 0.80) (Fig. 2A). There was no significant difference between the three different pairing combinations based on how the UHF coefficients were set for the interacting collars (*same setting-same setting, individual setting-same setting, individual setting-individual setting*) under the three treatments: no amalgamation (F_1,14_ = 0.28, *P*  = 0.76); amalgamation (F_1,14_ = 1.92, *P*  = 0.19); amalgamation and 1 s removal (F_1,14_ = 1.89, *P*  = 0.19).

There was a weaker, albeit still significant, agreement between the number of different contacts recorded by each collar and its reciprocal under all three treatment scenarios: no amalgamation (F_1,14_ = 9.00, *P*  = 0.01, *r* ^2^ = 0.41); amalgamation of those less than 1 minute apart (F_1,14_ = 11.63, *P*  = 0.005, *r* ^2^ = 0.47); and amalgamation and removal of any remaining 1 second contacts (F_1,14_ = 9.92, *P*  = 0.008, *r* ^2^ = 0.43). A one-way ANOVA showed that there was a significant difference between the three pairing combinations (*same setting-same setting, individual setting-same setting, individual setting-individual setting*) under two of the three treatments: no amalgamation (F_1,14_ = 4.82, *P*  = 0.03); amalgamation (F_1,14_ = 3.70, *P*  = 0.06); amalgamation and 1 s removal (F_1,14_ = 3.90, *P*  = 0.05). A Tukey’s post-hoc test showed that this difference was driven by collars set using the *individual-setting* method, which recorded a greater number of contacts than the collars set using the *same setting* method when paired together (All *P*<0.05, other pairing combinations, *P*>0.20; Fig. 2B).

### Validation in the Field

#### Accuracy of proximity logger identification

The cattle collars recorded a total of 1,290,632 interactions over a 12-month period, of which there were 471 records for spurious proximity logger identification codes. This represents an identification error rate for cattle collars of around 0.04%. These could be genuine interactions with deployed collars where for some reason the identification code was recorded incorrectly (perhaps due to interrupted signals or ‘data packet collisions’ when multiple packets of data arrive at the receiver due to simultaneous interaction between several animals) or erroneous records unrelated to any interaction. Badger collars recorded 308,318 contacts of which only three (0.001%) were deemed to be erroneous, with the ID of the individual contacted being a number that had not been deployed. The base stations recorded 5275 records, none of which had an obviously erroneous identification code. Taken together, these data suggest the identification error rate for all types of logger combined to be approximately 0.03%.

#### Cattle observation study

Of the 179 interactions observed during the six hours of focal observations, 129 (72%, range: 50%–94%) were recorded by the proximity loggers (Table 2). The median duration of the 50 interactions that were observed but not recorded by the loggers was 2 s (range: 1 to 80 s; mode: 1 s).

**Table 2:** Comparison of the observed number and duration of interactions.

Cattlenumber	Number of observed interactions	Number (%) of observed interactions recorded by proximity logger	Total durationof observedinteractions	Total durationof observed interactions recorded bythe proximity logger	Number (%) of observed interactions recorded by the proximity logger as split contacts
1	13	7 (54)	3∶46	1∶42	0 (0)
2	15	12 (80)	2∶42	3∶05	1 (7)
3	21	12 (57)	4∶39	3∶30	2 (10)
4	10	5 (50)	0∶49	0∶50	1 (10)
5	14	10 (71)	3∶39	4∶37	3 (21)
6	13	10 (77)	1∶21	2∶00	2 (15)
7	18	13 (72)	9∶35	7∶57	5 (28)
8	17	16 (94)	8∶15	8∶44	2 (12)
9	10	5 (50)	9∶34	5∶31	1 (10)
10	14	12 (86)	9∶17	7∶07	6 (43)
11	19	15 (79)	1∶30	1∶57	0 (0)
12	15	12 (80)	15∶51	9∶41	4 (27)
***Total number (mean %)***	***179***	***129 (72)***	***73∶58***	***56∶41***	***27 (15)***

This was done for 12 randomly selected cattle within a herd of 24 (focal observation sessions each lasted 30 minutes), with data recorded by proximity logging collars worn by the animals. Times are in minutes:seconds.

#### Cattle observation study: broken contacts

The cattle proximity loggers split 27 of the 179 records (15%) of observed interactions into multiple shorter records. The observed duration of the shortest interaction that was recorded by the loggers as multiple shorter records was 15 s (recorded as two 1-second interactions separated by a gap of 13 s), and all interactions lasting 14 s or less were recorded as single whole records. The longest interaction recorded as one complete record was 87 s. The longest interaction recorded as a split record was 313 s (recorded as four shorter interactions separated by three gaps). The median gap duration for split records was 20 s (range: 12 to 153 s; mode: 18 s). Of the interactions recorded as split records, the 95^th^ percentile for gap duration was 51 s. Thus on 95% of occasions, the maximum interval between logger records for interactions which were recorded as multiple shorter records was less than 51 s.

Of the 1,290,632 interactions recorded by the cattle collars, and the 308,318 interactions recorded by badger collars, over 58% (755 946 records), and 51% (151 076 records) respectively, were of 1 second duration. We investigated what effect omitting these records from the cattle proximity logger dataset had on that dataset’s similarity with the records from the cattle observational study. We did this both for dyadic interactions ‘as recorded’, and for combined dyadic records if they occurred within 51 s (the 95^th^ percentile for gap duration between pairs of cattle collars) of each other and involved the same two animals. First, observed records from all 12 loggers in the validation study were compared with proximity logger records without combining records less than 51 s apart and without filtering out 1 second contacts. The recorded dataset was significantly different to the observed contacts in the same time period (paired t-test, t  = 4.64, df  = 128, observed mean ± sd  = 31.0±50.7, edited mean ± sd  = 10.8±16.2, *P*<0.001). Second, observed records were compared with proximity logger records without combining records less than 51 s apart but this time filtering out all 1 second contacts. The recorded dataset was still significantly different to the observed contacts in the same time period (paired t-test, t_1, 89_ = 3.75, observed  = 36.2±55.1, edited  = 15.0±17.7, *P*<0.001). Third, observed records were compared with proximity logger records where dyadic records had been combined if they occurred less than 51 s apart, without filtering out 1 second contacts. The edited dataset was again significantly different to the observed contacts in the same time period (paired t-test, t_1, 127_ = 2.32, observed = 31.0±50.7, edited  = 26.4±46.8, *P* = 0.022). Finally, observed records were compared with proximity logger records in which dyadic records had been combined if they occurred less than 51 s apart, and then 1 second contacts were removed from the dataset. This time there was not a significant difference between the edited dataset and the observed contacts in the same time period (paired t-test, t_1, 89_ = 1.71, observed = 36.2±55.1, edited = 31.7±51.1, *P* = 0.09).

## Discussion

Proximity loggers are being increasingly used to study a wider range of free-ranging animal interactions. These devices have several user-defined parameters, the most important being detection distance and separation time, which allow them to be used in studies of different focal species and with very different aims. In the present study we have highlighted sources of inaccuracy on the basis of which we can propose unified methods for i) pre-deployment setting of proximity devices and ii) preparing derived data for analysis. In doing so we aim to improve the validity of data arising from the use of proximity loggers in future studies of animal contact networks, whilst at the same time recognising that there will always be limitations to the technology, for example due to the physics of UHF waves with which they operate.

The recording of erroneous data does not appear to be a significant problem with the latest generation of proximity logger, and can be considered to have a negligible impact on the data recorded. Based on the very small number of erroneous identification codes recorded, the proximity loggers appear to be extremely accurate in recording the identification of contacted collars. However, we were unable to determine what proportion of interactions recorded by the loggers as genuine identification numbers may in fact have been false. If a logger identification number existed then it was taken to be a true record. It was not possible to determine the ‘false record’ rate in the observational study as this would have required more accurate determination of separation distances than was achieved here.

The detection distance of all types of proximity logger reduced with time for those collars that had been deployed in the field, but not for the couple that had been kept in the laboratory with the battery turned off. Thus, rather than this being a feature intrinsic to the technology, it is more likely related to diminishing battery power. This in turn may be a function of temperature: at temperatures above and below 25°C, the voltage of lithium thionyl chloride batteries – as used in these proximity loggers – sags under load (K. Lay, *pers comm*). The proximity loggers fitted to the badgers are likely to have been exposed to warmer temperatures than the cattle loggers due to the sett environment and the closer fitting of collars to the badgers’ necks. The reduction in detection distance was very pronounced for the badger collars where a decline of almost 50% in detection range was observed over eight months, although there was no further decrease after 12 months of deployment when a critical battery threshold may have been exceeded. Also, it was not found to be influenced by the frequency or the duration of contacts that the collars had recorded. It therefore appears that longer range interactions are less likely to be recorded by the loggers over time, but that this decrease in detection distance levels off after eight months. A possible practical solution would be to periodically re-measure the detection ranges of the loggers and recalibrate as necessary. However, this could be difficult if a large number of loggers have been deployed and is likely to be highly impractical for loggers fitted to elusive wild animals that are not amenable to frequent recapture. An alternative solution would be to apply a correction factor to the data pre-analysis to account for the decrease in detection of longer range interactions over time and avoid biases in the interpretation of the data. Indeed, one general limitation of the technology at present is the requirement for animals to be recaptured in order to download the data stored in the internal memory. However, there is not a time limit for this and data can still be retrieved after the battery has run out (K. Lay, *pers comms*), although that situation was not encountered in this study.

Overall, the proximity loggers recorded a reasonable majority of the observed interactions although there was marked variation between individual loggers. The impact of missed interactions is likely to be very low because the modal duration of non-recorded interactions was 1 second, and all contacts of this duration were later filtered out of the dataset to improve reliability after combining “broken contacts”. Some interactions that were not recorded by the loggers were observed to be very close contacts – it was not just the longer-range interactions that were missed. The probabilities of detecting intraspecific contacts among white-tailed deer (*Odocoileus virginianus*) using proximity logger collars versus direct observation were quantified in a recent study where it was estimated that approximately 9% of contacts went unrecorded [19]. Non-recording of contacts might be due to the varied orientation of the loggers, or physical obstructions such as an animal’s head, vegetation, or nearby objects deflecting the loggers’ signals [9], [11]. For these reasons, some spatial imprecision and a small amount of nondetection bias is likely to remain a limitation of proximity logger use in the field.

The tendency of proximity loggers to record extended duration interactions as a series of shorter contacts has been reported previously [9], [11]. Not all subsequent studies appear to have accounted for this, and where they have, no consensus seems to exist on how to manipulate the data. Methods that have been applied include: joining contacts divided by periods less than the programmed ‘separation time’ [13], [16]; separation time plus 15 s [9]; scoring the length of contact as the union time between two collars [15]; aggregating records from single devices over a sliding window of 20 s [28]; and combining records detected within 60 s of each other [29]. The findings of the present study support the proposition of a 1 to 2 minute amalgamation window for records between pairs of loggers, since for 95% of the time where longer interactions were recorded as multiple shorter records, the gap between records was 51 s or less for the field trial and 129 s (just over 2 minutes) or less for the laboratory trial.

Proximity loggers that interact at the edge of their detection range may record very short contacts (typically of 1 second duration) possibly due to weak signal strength [9]. Removing these contacts from the dataset has been shown to have significant effects on contact network structure [15] and increases the reliability of pairwise contact records [9]. Despite this, removal of 1 second records has not been routinely conducted in all studies. The results of the present study indicate that proximity logger datasets should be filtered of 1 second records after combining dyadic records over a 1 to 2 minute amalgamation window, which produces a dataset that is closer to the observed values. Analysing unfiltered data may lead to erroneous conclusions; in most cases overestimating the frequency and underestimating the duration of contacts, which is also likely to impact the analysis of social networks and the metrics derived from the data.

The similar performance of the two methods for setting badger collar initiation distances (either a separate UHF setting for each collar [*individual setting*], or using the same coefficient for all collars [*same setting*]) suggests that it is not necessary to individually measure and set each collar to a particular UHF coefficient. An interesting result from this analysis is that interacting collars have a high level of agreement in the duration of the contacts that they record, but less of an agreement (albeit still significant) in the number of contacts that they record, suggesting that the length of the contact recorded may be a more accurate parameter to use in further analyses than the frequency of contacts recorded. It would be useful to investigate the influence of proximity logger separation time on data subsequently collected by loggers, since it may be expected that the longer separation times would result in fewer recorded interactions but those that were recorded would likely be of longer duration. We did not investigate this in the present study.

Taken together, the findings of this validation study can be summarised in a series of five recommendations which may guide researchers using proximity loggers to study animal contact behaviour in the future:

1. Assuming deployment is over a long period, consider setting proximity logger detection range slightly long at the start to compensate for the observed decrease in the initiation and termination distances of the collars. Alternatively, measure the detection distances of proximity loggers periodically and consider recalibrating every six months if practical, and consider incorporating a correction factor into data analyses if comparing across time periods (e.g. seasonal variation in behaviour).
2. When manipulating the data collected by automated proximity loggers, contacts recorded within 1–2 minutes of each other should be amalgamated if they involve the same pair of loggers. This will give a more accurate reflection of longer duration interactions, and can be easily automated, for example with a script in the statistical programme R (provided in Supplementary Material, along with a script for building symmetrical association matrices from raw proximity logger data – Document S1 and R Functions S1). When practically possible, we also encourage other uses of this technology to carry out similar trials to derive an amalgamation window for their devices.
3. Remove all records of interactions lasting 1 s from the dataset post-amalgamation, as these may represent weak signals or collars interacting at the edge of their detection range and their removal increases the accuracy of the dataset. Whilst other studies have reported this, ours is the first to explicitly test how the resultant data matches ‘real-life’ data.
4. Include VHF transmitters in all proximity logger devices to increase recovery rate if collars fall off or if base stations go missing.
5. As some proximity loggers are unlikely to be recovered from the field, based on the losses encountered in the present study, we suggest budgeting for 110% of the required number of large animal (in this case, cattle) loggers, 150% for medium-sized highly mobile animal (in this case, badgers) collars, and 175% for static base stations. These budgets should be taken as a guide rather than being prescriptive because rates of collar loss are likely to differ amongst species and users.

In conclusion, this study indicates that proximity loggers are highly accurate at recording the identification of contacted loggers but less reliable at consistently determining the true frequency and duration of contacts. Our investigations of these limitations in proximity logger performance have allowed us to quantify these sources of potential error and to suggest approaches for their mitigation. We hope that the five recommendations made here will be of use to the expanding number of researchers using proximity loggers to determine contact patterns of animals and provide an evidence base on which data collected from these devices may be corrected to more accurately reflect the ‘true-life’ pattern of animal interactions.

## Supporting Information

Document S1 **A guide to the use of the two Functions for which the R code is provided.**
(DOC)

R Functions S1 **R Code for the two Functions that can be used to filter and construct association matrices from data collected by proximity loggers.**
(R) Click here for additional data file.
