# Supplementary material for: Performance of Proximity Loggers in Recording Intra- and Inter-Species Interactions: A Laboratory and Field-Based Validation Study
Source: PLoS One. 2012 Jun 26;7(6):e39068. doi: 10.1371/journal.pone.0039068 (PMC3383709; doi:10.1371/journal.pone.0039068)
Supplement: Document S1 — A guide to the use of the two Functions for which the R code is provided. (DOC) [file pone.0039068.s001.doc]

**Supporting Material**

**Performance of proximity loggers in recording intra- and inter-species interactions**

**Drewe J, Weber N *et al.* *PLoS ONE***

The following is a guide to the R functions that accompany the Drewe *et al.* paper regarding proximity logger performance. For any queries about these functions, or bug reporting, contact Xavier Harrison, [xav.harrison@gmail.com](mailto:xav.harrison@gmail.com)

# Raw Data

Both functions assume data are in the following format. Column names have to be as listed, otherwise the functions will throw an error. Column order must also be preserved.

Variable Descriptions

**Record:** unique record id logged by the loggers

**Encounter**: unique code of the other collar encountered

**Date**: calendar date of contact. Must be in the format ‘dd/mm/yyyy’.

**Time** is the start time of the contact. Must be in format ‘HH:MM:SS’

**Length:** Duration, in seconds, of the contact.

| **record** | **encounter** | **date** | **time** | **length** |
| --- | --- | --- | --- | --- |
| 240 | 31 | 21/05/2009 | 03:48:57 | 1 |
| 241 | 31 | 21/05/2009 | 03:50:12 | 1 |
| 242 | 31 | 21/05/2009 | 03:51:28 | 1 |
| 243 | 31 | 21/05/2009 | 03:56:54 | 1 |
| 244 | 31 | 21/05/2009 | 03:58:14 | 673 |
| 245 | 31 | 21/05/2009 | 04:15:15 | 354 |
| 246 | 31 | 21/05/2009 | 04:43:00 | 1 |

# ‘Contactweld’ Function

**Function designed to combine broken contacts that fall within a user-specified time limit into one contiguous record.**

Step by Step Guide

1. Read the data table into R in the above raw data format.

2. The ‘contactweld’ function takes two arguments. Firstly the name of the dataframe containing the raw data, and then the time difference (in minutes) that will decide if records will be combined.

R Code:

newdata<-contactweld(dataframe,5)

In general, if *t* minutes are specified, records of length equal to, or less, than *t**60 seconds will be combined. Therefore, if record *x+1* starts within *t* minutes of record *x* ending, these two will be combined. If record *x+2* also starts within *t* minutes of *x+1* ending, all three will be combined. In this case the ‘start’ time of record *x* will be taken as the start, and the ‘end’ time of record *x+2* taken as the end, and the time difference between these two times calculated.

**Output:**

Columns 1-5 of the output will be exactly the same as the input dataframe (Raw Data), which may be useful for error checking. The new column ‘contact’ is the corrected contact time between two individuals. There is also an ‘end’ time column which one might find useful, but this is a combined date and time object so if it is exported to a spreadsheet editor one might wish to split the columns so that the date and time can be manipulated separately.

**Troubleshooting**

If the function throws an error, it is most likely one of two things: Either the column names aren’t exactly as above, or the date is in a format that R the function doesn’t recognize.

# ‘Matrixbuild’ Function

**Builds a symmetrical association matrix from raw proximity logger data. Matrix options include specifying a matrix of Total Contact Duration or Total Frequency of Contact Events, as well as being able to only select records falling within a specified timeframe (e.g. only records from 5pm to 10pm). Matrices built by this function can be fed directly into social networking software like UCINET and SocProg.**

**The function automatically selects the MAXIMUM datum recorded between pairs of individuals. This can occur because two loggers within the threshold proximity should theoretically record the same duration of contact, but often will not. In cases where this discrepancy occurs, the largest value will always be the one to appear in the output matrix. The matrix will also be symmetrical in that the lower half will be a perfect reflection (duplicate) of the upper half.**

**N.B. This function incorporates the ‘contactweld’ function listed above, and so ‘contactweld’ must be loaded in order for ‘matrixbuild’ to work. It is best to familiarize yourself with the contactweld function prior to using ‘matrixbuild’.**

Step-by-Step Guide

1. Set working directory where the individual data files are located.

The function assumes each individual has its own .txt data file and that all these files are stored in the same folder. Names of data files must start with the unique animal ID, but may contain suffixes. ‘008p.txt’ and ‘008pWinter.txt’ are both fine.

1. Make a vector in R containing a list of all the filenames. Each one has to be in quotes, but you don’t need the file extension (like .txt) e.g.

R Code:

listofanimals<-c(‘008pwinter’,'019psummer','010y','011y', '016p','018p','020y'…)

1. Make a 2-column dataframe containing unique collar IDs (see ‘Raw Data’ section) in the first column, and the unique animal ID in the second column. The function will use these data to match the logged ID in the raw data to the individual wearing that collar. The IDs in column 2 will be the IDs that appear in the final contact matrix and MUST match the IDs found at the start of the list of filenames in ‘listofanimals’(see above).

This dataframe should contain information on ALL individuals/collars that have been deployed. This is because you may read in data files for 30 individuals, but these files may contain contact information from >30 proximity loggers. Such situations arise when loggers are deployed but lost/never retrieved. These individuals must be represented here so that they appear in the final matrix.

| ID | Individual |
| --- | --- |
| 1 | 008p |
| 2 | 067y |
| 3 | 045p |
| 4 | 050y |
| 5 | 063p |
| 6 | 043w |
| 7 | 082w |
| 8 | 062p |
| 9 | 015p |
| 10 | 012b |

**Table 1**. Example table of unique proximity logger IDs (ID) and the corresponding unique animal ID (Individuals). Column headings can be named arbitrarily, as long as the column order is preserved.

The dataframe in table 1 can be constructed in R with the following code. However large tables are more efficiently loaded directly from an eternal table.

R code:

collarids<-data.frame(ID=seq(10),Individual=c(‘033p’,’067y’,’045p’…))

1. Then, simply pass the vector of filenames and the dataframe of IDs to the ‘matrixbuild’ function:

R Code:

newmatrix<-matrixbuild(listofanimals,collarids)

1. Firstly, the function will ask for the number of characters required to extract the animal IDs from the filenames. Thus, if the file was called ‘008pwinter’, entering ‘4’ will extract ‘008p’, which matches the ‘Individual’ in row 1 of the ‘collarid’ dataframe. It is important to always make sure that the IDs can be matched in this fashion.

N.B. If unique animal IDs (column 2) are of differing length (e.g. “Jake”,”Bob”,”Jo”) it is advised to pad the filenames with trailing underscores (“Jake”,”Bob_”,”Jo__”) so that all IDs are extracted correctly with a single argument for number of characters.

1. The function will prompt you with the name of the working directory from where the datafiles will be loaded. If the directory is correct, enter ‘Y’ or ‘y’. If you enter ‘N’ or ‘n’ the function will terminate so that you can change the working directory.
2. If you entered ‘Y’, the function will tell you how many datafiles were loaded successfully. This should be the same as the number of individuals in the ‘listofanimals’ vector that was passes to the function.
3. You will then be prompted for the threshold number of minutes to be used by the ‘contactweld’ function to join broken contacts together (e.g. 5 minutes). So if you enter ‘5’ (no quotes) the function will run the contactweld function for each of the datafiles in turn using this threshold.

NB. If you do not wish to use the contactweld function (i.e. build the contact matrix directly from the raw data, simply enter ‘0’ (zero)

1. An output will inform you that the contactweld function is running for each of the *n* data files. Depending on the number and size of raw data files, this may take some time (1-2 minutes).

R Output:

[1] Running contactweld Function for 37 datafiles using threshold of 5 minutes

1. You will be asked if you wish to subset the data based on the time of the records. If you specify ‘N’, the function will move onto Step 12, otherwise you will be asked for the start and end time of the specified time window, in 24h format (e.g. 17 for 5pm, or 10 for 10am). Note that currently the function only supports subsetting to units of 1 hr i.e. not to the resolution of minutes.
2. Output will confirm the time window which you have specified e.g. if one specified ‘10’ for start time and ‘12’ for end time, output will show:

R Output:

[1] Subsetting Records to Those Between 10:00 and 12:00

1. At this step you can specify the metric you desire: either ‘D’ for total summed contact duration between individuals, or ‘F’ for frequency (count) of contact events, irrespective of duration. These metrics are calculated AFTER the ‘contactweld’ function has run.
2. Output will then inform you of the dimensions of the matrix being built. If some loggers have not been retrieved, the dimensions will typically be larger than the number of datafiles initially supplied.

R Output:

[1] Building 44 x 44 Matrix...

1. Assuming no errors, you will be informed that the matrix has been built successfully, as well as the largest contact (frequency or duration, depending on specified option)

R Output:

[1] Matrix Built Successfully...Longest Contact is 812.3 minutes

1. If you have specified time subsetting, it is possible that some of the *n* files read into R may contain no records in the specified window. If this is the case, you will be informed of a) the number of files with no records and b) the IDs of the individuals:

R Output:

[1] N.B. 2 datafiles have no records in the specified timeframe

[1] Animal IDs 013p,032y have no contacts

1. You will then be prompted for the name of the output file. Entering ‘Matrix1’ (no quotes), will create a file called Matrix1.csv in the working directory that can then be opened with a spreadsheet editor. The matrix will also be available to manipulate in R, as long as it is ‘assigned’ using ‘<-‘ e.g. in this example an object called ‘newmatrix’ will be created in R.

R Input:

newmatrix<-matrixbuild(listofanimals,collarids)

**END**
